# Regulative effects of curcumin spice administration on gut microbiota and its pharmacological implications

**DOI:** 10.1080/16546628.2017.1361780

**Published:** 2017-08-09

**Authors:** Liang Shen, Lu Liu, Hong-Fang Ji

**Affiliations:** ^a^ Shandong Provincial Research Center for Bioinformatic Engineering and Technique, School of Life Sciences, Shandong University of Technology, Zibo, PR China

**Keywords:** Curcumin, gut microbiota, regulation, mice

## Abstract

Curcumin, the major active component of turmeric (*Curcuma longa*), is widely used as a spice and food-coloring agent, and also exhibits multiple biological activities. However, as curcumin has poor systemic bioavailability its pharmacology remains to be elucidated. Owing to the high concentration of curcumin in the gastrointestinal tract after oral administration, we hypothesize that it may exert regulative effects on the gut microbiota. We investigated the regulative effects of oral curcumin administration on the gut microbiota of C57BL/6 mice and found that curcumin significantly affected the abundance of several representative families in gut microbial communities, including Prevotellaceae, Bacteroidaceae, and Rikenellaceae. Considering the pathogenic associations between gut microbiota and many diseases, the present findings may help us to interpret the therapeutic benefits of curcumin.

## Introduction

Curcumin is a major effective component of dried rhizomes of *Curcuma longa* and is widely used as a food-coloring pigment and a preservative. Curcumin is also a highly pleiotropic natural polyphenolic compound possessing numerous pharmacological activities and therapeutic potential against many diseases [1–5]. In spite of its therapeutic potential against a wide spectrum of human ailments, curcumin has poor systemic bioavailability, as demonstrated by many clinical studies [1,^6^,^7^]. Even after high oral doses (up to 8 g/day), serum levels of curcumin were undetectable in humans [8]. The poor systemic bioavailability of curcumin not only mystifies its pharmacology, but also largely limits its clinical application.

In recent years, an exponentially increasing number of studies has indicated that the alterations in the intestinal microbiota are linked with many metabolic diseases, including obesity, diabetes, and chronic liver disease [9–12], and the intestinal microbiota is proposed to be a novel potential therapeutic target for these microbiota-associated diseases. Although curcumin has poor systemic bioavailability, after oral administration it is expected to be present in high concentrations in the gastrointestinal tract. Thus, it is rational to infer that curcumin may exert direct regulative effects on the gut microbiota. This may be an important mechanism underlying its therapeutic benefits [13] and could explain the paradox between curcumin’s poor systemic bioavailability and its widely reported pharmacological activities. Thus, the current study was designed to explore the regulative effects of oral administration of curcumin on the gut microbiota of mice, to provide deeper insights into the pharmacology of this natural compound.

## Materials and methods

Curcumin, a natural mixture isolated from turmeric, containing the three main components of curcumin 40.9%, demethoxycucumin 33.2%, and bidemethoxycurcumin 23.3%, was purchased from Shanghai Macklin Biological Technology Co. (Shanghai, China). Carboxymethylcellulose sodium salt (CMC-Na) was obtained from Sigma-Aldrich Trading Co. (Shanghai, China). Curcumin was dissolved in 0.5% CMC-Na before oral administration to mice. Twelve 3-month-old C57BL/6 mice (obtained from Vital River Laboratory Animal Technology Co., Beijing, China) were randomly separated into two groups and housed under specific-pathogen-free conditions. The mice were fed a standard chow diet. The curcumin-administered group (*n* = 6) received curcumin gavage in a dose of 100 mg/kg body weight. The continuous once-daily oral administration of curcumin was given for 15 days. The control group (*n* = 6) was supplied with the same feeds but without curcumin gavage.

Fresh fecal samples were collected into sterile Eppendorf tubes and frozen immediately at –80ºC until DNA extraction. The extracted DNA from each fecal sample was used as a template to amplify the V3 and V4 regions of the bacterial 16S ribosomal RNA gene. Amplicon sequencing libraries were sequenced on the Illumina Miseq platform for paired-end reads of 300 bp. Several *α*-diversity indices, including Chao1, PD_whole_tree, Shannon, and Simpson, were calculated to evaluate the richness and diversity of the gut microbiota. The animal studies were approved by the Animal Use Subcommittee at the Shandong University of Technology.

## Results

**Figure 1.**
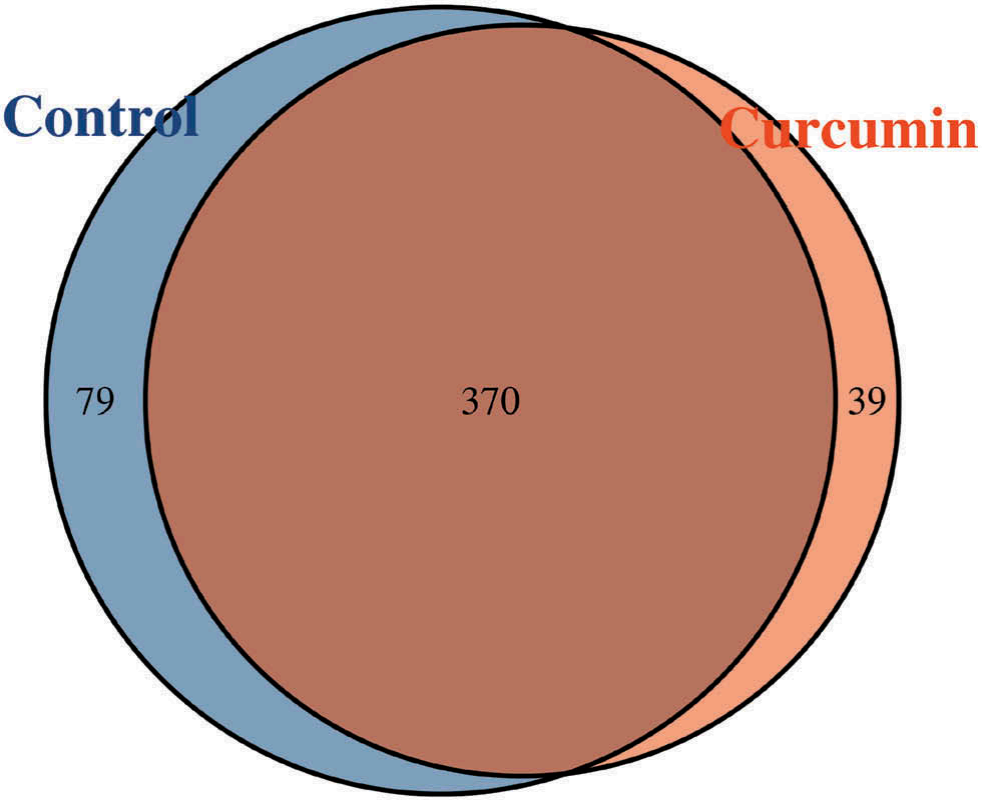
Venn diagram of the shared operational taxonomic units in the curcumin-treated and control groups.

Paired-end sequencing of the V3–V4 regions of 16S ribosomal DNA genes was implemented on the 12 samples. In total, 333,936 usable reads (27,828 per sample, read length = 220–500 nt) were obtained from 12 samples. Altogether, 488 operational taxonomic units (OTUs) were displayed at the 97% similarity level. According to the Venn diagram showing the shared OTUs between the curcumin-administered and control groups (Figure 1), there was a total of 370 shared OTUs between the curcumin and control groups, and 39 were unique in curcumin group and 79 in the control group.

To explore the effect of curcumin treatment on the richness and diversity of the gut microbiota, we compared the *α*-diversity metrics (including Chao1, PD_whole_tree, Shannon, and Simpson) of the control and curcumin-administered groups. According to the data in Table 1, as *p* > 0.05 for all indices, it was deduced that curcumin administration tended to decrease the microbial richness and diversity, while there were no significant differences between the control and curcumin groups.

**Table 1.** Gut microbiota diversity of the control and curcumin-treated groups of C57BL/6 mice.

Group	Chao1	PD_whole_tree	Shannon	Simpson
Control	318.20	19.45	5.74	0.96
Curcumin	282.98	16.94	5.73	0.96

**Figure 2.**
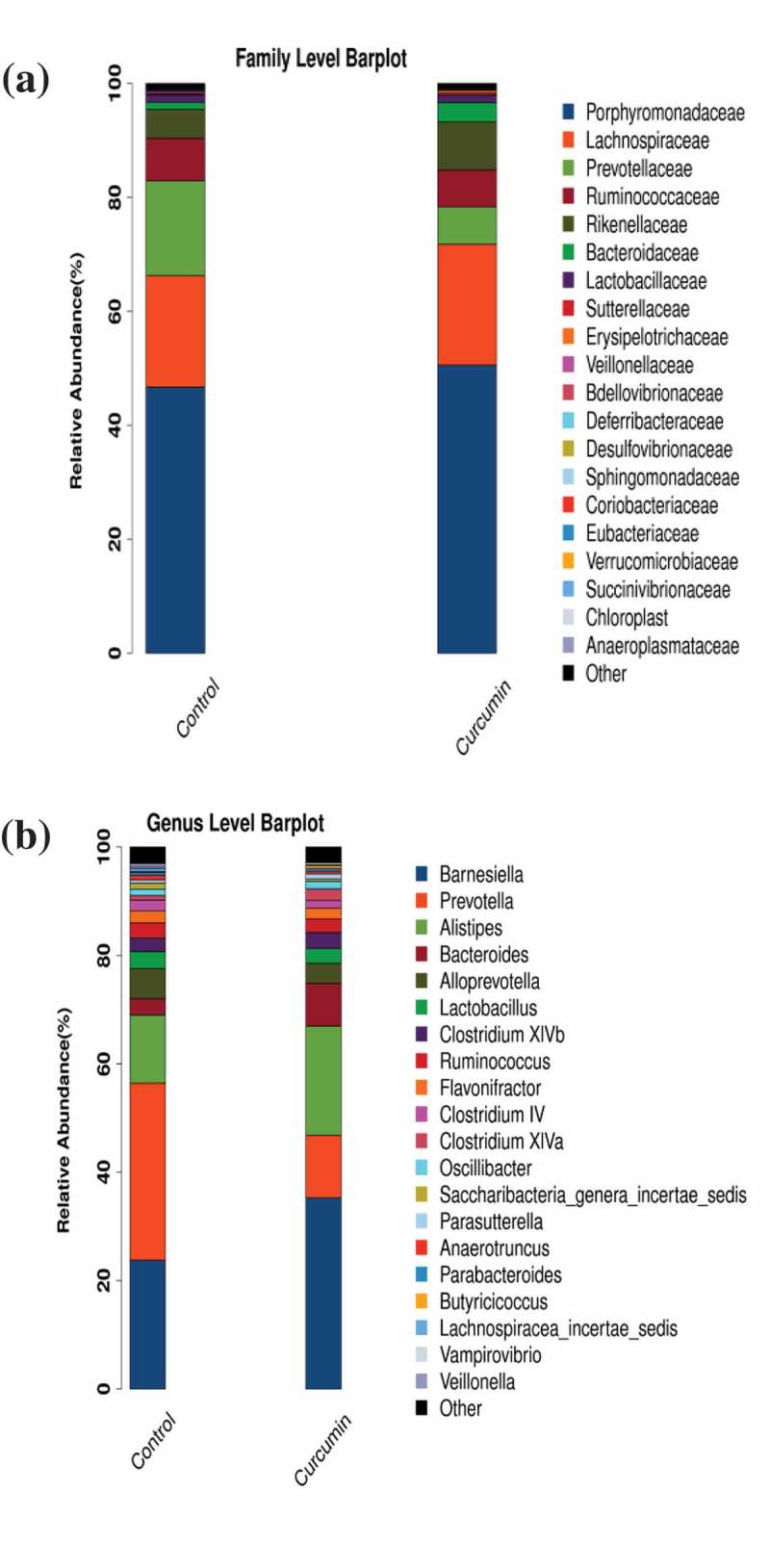
Structural comparison of gut microbiota between the curcumin-treated and control groups at (a) family and (b) genus levels.

To investigate in detail the regulative effect of curcumin on gut microbiota, we then compared the bacterial composition between the curcumin-administered group and the control group at the family and genus levels. At the family level, there were four families in total with significant differences in abundance (*p* < 0.05) between the curcumin and control groups. Figure 2(a) shows the 20 most abundant bacterial families in the curcumin and control groups. The abundance of Prevotellaceae decreased significantly in the curcumin group relative to the control group, from 15.48% to 6.16% (*p* = 0.01). In comparison, the abundance of Bacteroidaceae in the curcumin group (3.21%) was significantly higher than in the control group (1.15%, *p* = 0.00). The abundance of Rikenellaceae also increased significantly, from 7.96% to 4.73% (*p* = 0.04).

At the genus level, a total of four genera exhibited significant differences in abundance between the curcumin-administered group and the control group. The 20 most abundant genera in the two groups are shown in Figure 2(b). A significant reduction in *Prevotella* abundance, from 13.29% to 4.63% (*p* = 0.00), from the control group to the curcumin group was observed. There was a significant increase in *Alistipes* abundance, from 4.73% to 7.96% (*p* = 0.04). Similarly, the abundance of *Bacteroides* in the curcumin group (3.21%) was significantly than in the control group (1.15%, *p* = 0.00).

## Discussion

Curcumin, a natural polyphenolic compound contained in the spice turmeric, has diverse pharmacological effects. To exert these effects, curcumin should possess high *in vivo* concentrations; however, as it has been reported to have rather poor systemic bioavailability, its pharmacology remains to be elucidated. In view of its expected high concentration within the gastrointestinal tract after oral administration, curcumin may exert regulative effects on the gut microbiota. This motivated us to perform a comparative analysis on the gut microbiota in curcumin-administered mice and controls by pyrosequencing the V3 and V4 regions of the bacterial 16S ribosomal RNA genes. Despite no significant difference being found, oral administration of curcumin tended to decrease the microbial richness and diversity, which is consistent with the results of a study on the modulatory effect of curcumin on gut microbiota in a rat model of non-alcoholic fatty liver disease [14]. Curcumin administration affected the abundance of several representative families in gut microbial communities of C57BL/6 mice, including Prevotellaceae, Bacteroidaceae, and Rikenellaceae. Considering the dramatically increasing number of reports supporting the pathogenic associations between gut microbiota and many diseases in recent years, especially the significant changes in abundance of particular bacterial species in these diseases [15–21], the regulative effects on the gut microbiota may account in part for the therapeutic benefits of curcumin.

In addition, on the basis of the low stability of curcumin, previous studies supported that the degradation products make an important contribution to the pharmacological effects of curcumin [22–25]. At the same time, the gut microbiota can also transform curcumin [26,27]. It has been reported that microbial metabolism of curcumin with *Pichia anomala* yielded four major and two minor metabolites [26]. The two pathways, i.e. the contribution of the bioactive degradation and microbial metabolism products of curcumin, and the regulative effects on the gut microbiota, shed light on the complex pharmacology of curcumin due to its poor bioavailability. This study demonstrated the regulative effect of curcumin administration on gut microbiota in mice. More studies are required to extend the current gut microbiota outcomes found in mice to human studies, to provide a basis for gut microbiota-based therapeutic applications of curcumin.
